# Erratum: Strategical incoherence regulates cooperation in social dilemmas on multiplex networks

**DOI:** 10.1038/srep13511

**Published:** 2015-09-02

**Authors:** Joan T. Matamalas, Julia Poncela-Casasnovas, Sergio Gómez, Alex Arenas

Scientific Reports 5: Article number: 951910.1038/srep09519; published online: 04272015; updated: 09022015.

In the Supplementary Information file originally published with this Article, there are typographical errors.

In the section under ‘Convergence’,

“In order to evaluate such convergence, we fit the last *tγ* time steps of the evolution to a linear trend, 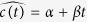 using the QR decomposition method”

should read:

“In order to evaluate such convergence, we fit the last *tγ* time steps of the evolution to a linear trend, 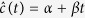 using the QR decomposition method”

In the section under ‘Analysis of Fluctuations’,

“For each one of the *I* repetitions of the experiment we fit a linear model to the final *tγ* time steps of the simulation, 
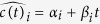
.”

should read:

“For each one of the *I* repetitions of the experiment we fit a linear model to the final *tγ* time steps of the simulation, 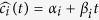.”

Lastly, Equation (2.1),


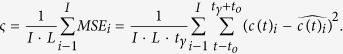


should read:


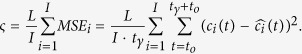


These errors have been corrected in the Supplementary Information that now accompanies the Article.

