# Supplementary material for: Strategical incoherence regulates cooperation in social dilemmas on multiplex networks
Source: Sci Rep. 2015 Apr 27;5:9519. doi: 10.1038/srep09519 (PMC5386185; doi:10.1038/srep09519)
Supplement: Supplementary Information [file srep09519-s1.pdf]

Strategical incoherence regulates cooperation in social dilemmas on multiplex networks

Joan T. Matamalas,[1] Julia Poncela-Casasnovas,[1] Sergio Gómez,[1] and Alex Arenas[1]

[1] *Departament d'Enginyeria Informàtica i Matemàtiques, Universitat Rovira i Virgili, 43007, Tarragona, Spain.*

I. CONVERGENCE

We study the system's convergence to the stationary state. It is well known that, in general, the time evolution of cooperation on a monoplex network, for a value of the parameters that allows the survival of at least some cooperation, usually follows a curve that initially decreases moderately, while cooperation rearranges itself from the random initial conditions into a more favorable setting (either in one or multiple clusters), and then there is a new increase, followed by the achievement of the stationary state. In general, this whole transient time is relatively short (typically of $1 - 2 \times 10^4$) for a size of $1 - 4 \times 10^3$ nodes. However, it hasn't been explored in detail until now the convergence process for the four games in the $T - S$ plane on multiplex networks.

In order to evaluate such convergence, we fit the last t_γ time steps of the evolution to a linear trend, $\widehat{c}(t) = \alpha + \beta t$ using the QR decomposition method. Then we use the slope of the fitted model to compute the variation of the density of cooperators every 1000 time steps, $\Xi = 1000 \cdot \beta$. Thus, a near-zero value of this metrics indicates that the system has reached the stationary state, while a positive value would indicate that the average level of cooperation is still increasing in the system at that time, and vice versa. Figure 1 shows how every point of the $T - S$ plane performs on our measure of convergence during the last t_γ time steps of the simulation.

Monoplex networks (left column in Figure 1) seem to reach the stationary state according to our convergence criteria for every point of the plane $T - S$ and independently of the initial fraction of cooperators: the slope is in general smaller than 10^{-4}. We observe, however, a small amount of stochastic noise for some regions of Snow-Drift and Prisoner's Dilemma games, where our measure indicates that the stationary is not fully reached. Nonetheless, we will show in Section II that this noise is just an effect of the large fluctuations in the number of cooperators when the stationary is reached.

In multiplex networks, on the other hand, there is a non-negligible area where convergence is not reached (red areas in the central and right panels in the first row in Figure 1). In the most extreme cases, where the slope of the linear model β is largest, our measurements indicates an increment of the cooperators of about a 0.1% every 1000 time steps. That could seem a smaill increase in the fraction of cooperators, however if the evolutionary process were to run for a very long period of time, the increase could be significant.

To better illustrate this difference in the path to stability for monoplex vs. multiplex networks, we show in Figure 2 the time evolution of the level of cooperation, $\langle c \rangle$ for a single simulation progresses (monoplex plotted in red and two multiplex networks with different number of layers represented in green and blue), for one point in the plane $T - S$. This particular point has been picked as an extreme case, for having the maximum fluctuation values in the entire $T - S$ plane (see Figure 3 and Section II for further detail). We clearly observe that, while the time required for the monoplex system to achieve the stationary state is around $1 - 2 \times 10^4$, for the multiplex networks it can be at least one order of magnitude larger, and it increases with the number of layers, too. However, it is important to remember that this example shown here is a very extreme case, while the convergence process in multiplex is in general faster for regions of the plane that are far away from the transition area (or areas where the final state is close to an all-cooperation or all-defection).

To understand the reason for such an increase in the convergence time for multiplex with respect to monoplex (at least in some regions of the $T - S$ plane), one has to pay attention to which areas are more reluctant to reach stability. Such regions correspond again to the transition areas between those that end up in total cooperation and those that end up in total defection. In the Stag Hunt quadrant, the game has an unstable equilibrium with mixed population, which means that the game will tend to converge to total cooperation or total defection as happens in the monoplex network. However the multiplex structure of the network changes that outcome, as we described in the Results Section. In these structures, the transition region is larger than in the monoplex, and is in this transition region where the convergence is hard. The analysis of how the fraction of cooperators has an effect on the convergence gives us an insight about what is happening. We have already stated that the interlayer dynamics has an important role in the survival rate of defectors and cooperators. If we look at the multiplex columns of the Figure 1, we can observe how the convergence is strongly affected by the initial fraction of cooperators. On the one hand, if the initial fraction of cooperators is small, they will need more time to reach the equilibrium because they have to fight against a larger fraction of defectors that benefits from the interlayer dynamics. On the other hand, a larger initial number

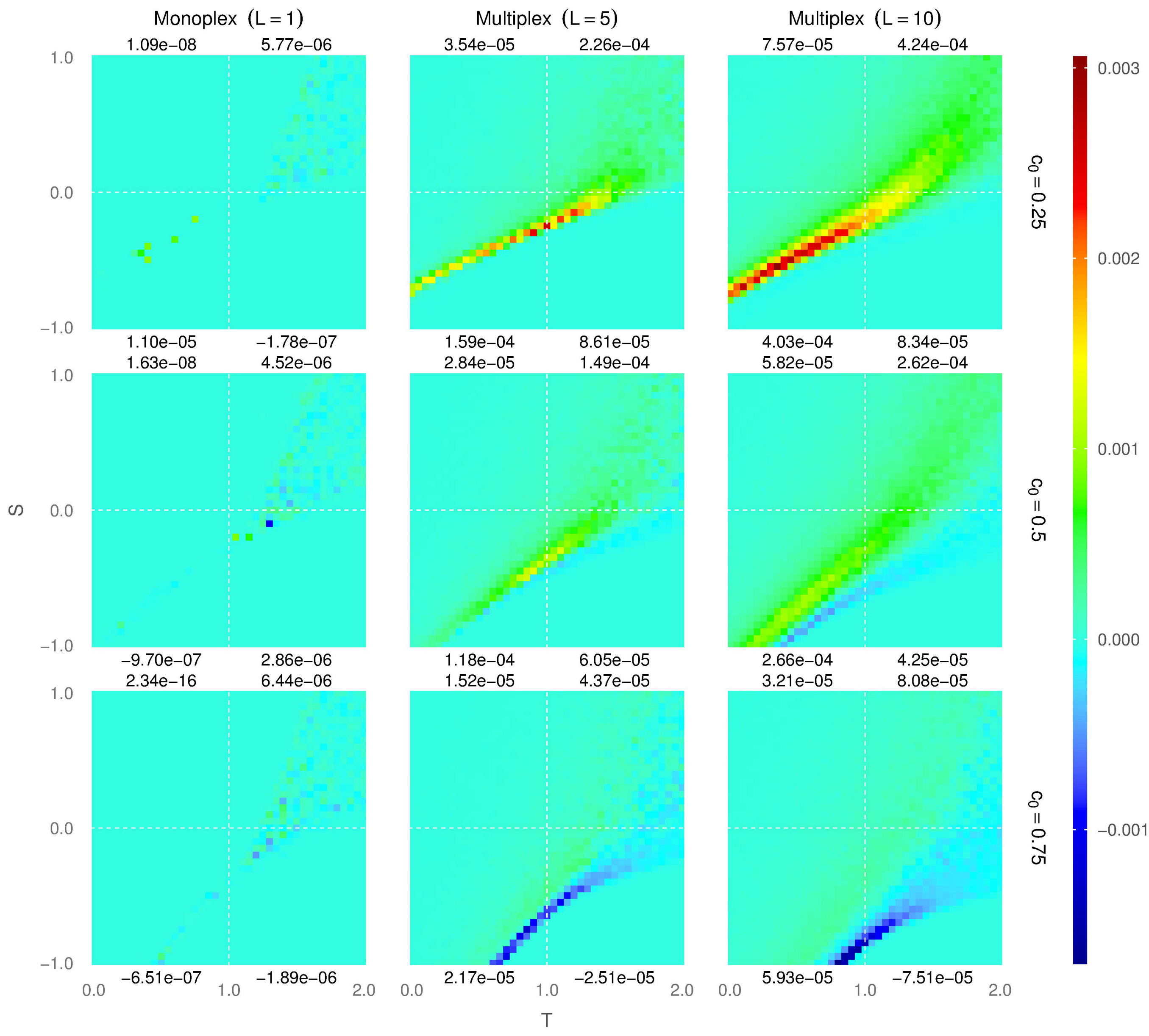

FIG. 1: Convergence to the stationary state measured as the average variation in the fraction of cooperators during 1000 time steps, measured using the slope of a linear model fitted at the end of t_γ steps of the simulation. The numbers in each quadrant represent the mean convergence value (the slope of the fit) for each one of the four games (upper-left is the Harmony Game, upper-right is the Snow Drift, Stag-Hunt is the lower-left, and the Prisoner's Dilemma in the lower-right). In the different rows we show the information for several values of initial fraction of cooperators ($c_0 = 0.25$, $c_0 = 0.50$, $c_0 = 0.75$), while the different columns correspond to 1, 5 and 10 layers, respectively.

of cooperators implies that the defectors will need more time to reach an stable configuration. However, the presence of a large number of initial cooperators has less impact on the convergence; which is easily understood, given the fact that defectors get more profit from cooperating in other layers than the opposite case.

Similar conclusions could be reached for the other games, taking into account that the transition regions between full-cooperation and full-defection are different in nature, for instance in the Snow-Drift this region is wider. Thus, we can see the effect of non-convergence is diluted across the Snow-Drift quadrant.

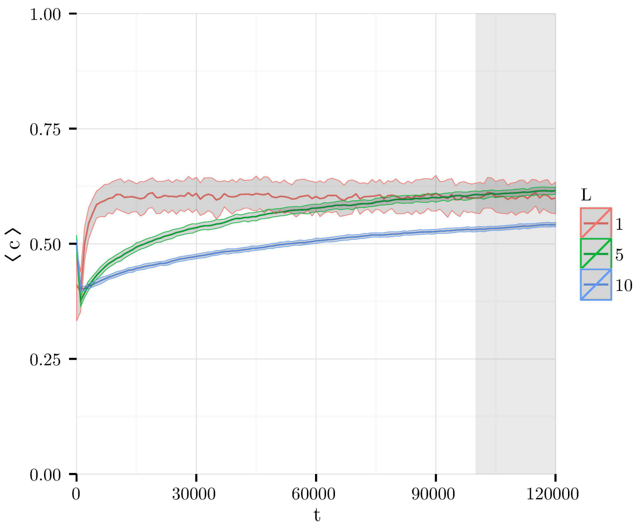

FIG. 2: Example of the time evolution of cooperation for the point in the plane $T-S$ with maximum fluctuations values ($S=0$ and $T=1.35$). The shadowed area for each plot represents two standard deviations over the residuals of the I iterations at each time step. It will be used to compare the size of the fluctuations between monoplex and multiplex networks. The grey vertical area corresponds to the interval $[t_0, t_0+t_\gamma]$ where the measures shown in all panel figures in this paper are computed.

II. ANALYSIS OF FLUCTUATIONS

We turn our attention now to the fluctuations of the system in the stationary state. In the case of these four games on a monoplex network, it is well known that the level of cooperation in the stationary state fluctuates around a well-defined average value due to the effect of both the topological structure of the network and the nature of the Replicator updating rule. We propose a measure in order to quantify these fluctuations and later compare them with the cases of multiplex networks. For each one of the I repetitions of the experiment we fit a linear model to the final t_γ time steps of the simulation, $\widehat{c_i}(t) = \alpha_i + \beta_i t$. We also need to take into account a possible non-zero slope in the measure of fluctuations (see in Section 1), so we average the Mean Square Error between the data from the simulations and the predictions of the linear model for the I iterations, calculated as:

$$\zeta = \frac{L}{I}\sum_{i=1}^{I} MSE_i = \frac{L}{I \cdot t_\gamma}\sum_{i=1}^{I}\sum_{t=t_0}^{t_\gamma+t_0}\left(c_i(t) - \widehat{c_i}(t)\right)^2. \tag{2.1}$$

The results for monoplex and multiplex networks are displayed in Figure 3. For the monoplex case, the simulations show small fluctuations in the quadrants of Stag-Hunt and Harmony Game. However for the Snow-drift on monoplex, the results display a zone where fluctuations are larger than in the rest of the plane $T-S$. That can be attributed to the nature of the game: it has an evolutionary stable equilibrium with mixed populations, so a consensus where both strategies coexist has to be reached. To achieve this objective some nodes have to alternate their strategies. These changes, due the topological features of the network, can lead to a cascade effect of changes in a large portion of the network; the equilibrium gets disturbed, and a new equilibrium has to be reached again. This causes the relatively large fluctuations that we measure. It is worth noticing that in the area of mutual coexistence of strategies the fluctuations are larger where the temptation and sucker payoffs are not far from the payoffs of mutual cooperation and mutual defection. It is also noteworthy that, even when the Prisoner's Dilemma quadrant presents very small fluctuations in general, it does show a small but very significant spot near the line of weak Prisoner's Dilemma, where they are large. Again, this corresponds to the area of competition between Cooperation and Defection, where each of the strategies accounts roughly for half the population.

The introduction of multiplex networks has an enormous effect on the fluctuations. The fluctuations are again in the region of coexistence of strategies, however, in the case of 5-layer multiplex the fluctuations are much smaller than in the monoplex case. The results in the 10-layer multiplex display an even larger reduction in the measure of fluctuations (compare also the three example curves shown in Figure 2). The nature of such reduction from monoplex to multiplex is to be found in the interlayer dynamics. Each layer is pushed to reach an stable equilibrium where both strategies can coexist, nonetheless the shared information between the layers establishes a way to constrict the

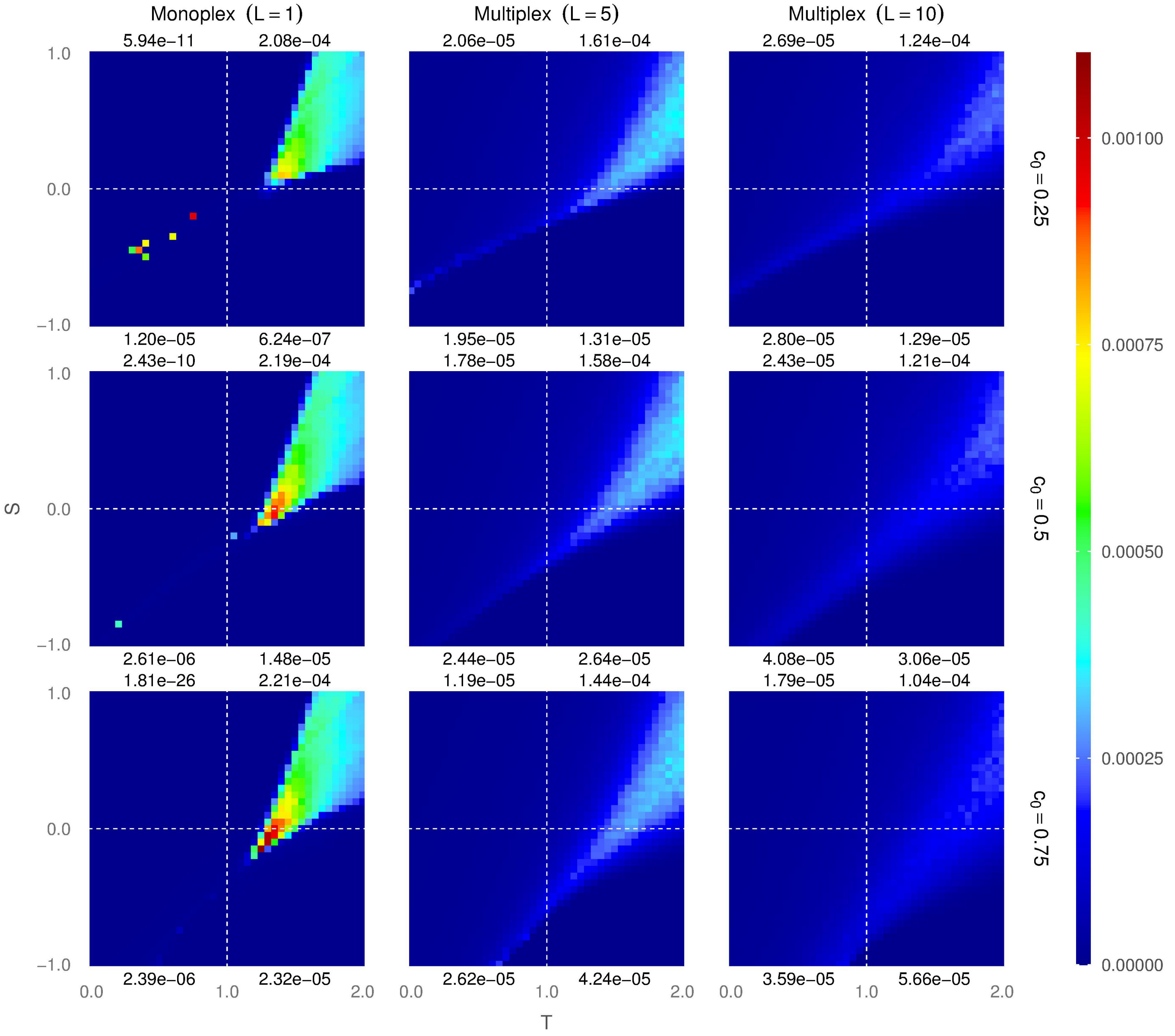

FIG. 3: Fluctuation of the fraction of cooperator around the fitted trend at the final time steps of the simulation computed as described in the equation (2.1), for each pair of values S and T. The number in each quadrant represents the average value for each one of the four games (upper-left is the Harmony Game, upper-right is the Snow Drift, Stag-Hunt is the lower-left, and the Prisoner's Dilemma in the lower-right). The results are provided by 3 different initial conditions

range of the fluctuations. The change of strategy of a node in one layers is not conditioned by its performance in that single layer, but by its global performance in the entire multiplex structure. That makes the system more robust to fluctuating nodes, at the expense of convergence time to the stationary equilibrium. We observe that, both for monoplex and multiplex structures, the initial fraction of cooperators, c_0, barely has any observable influence on the size of the fluctuations.

It is worth mentioning that the fluctuations shown in Figure 2 are calculated with a modified version of equation (2.1), as follows: we have to divide the time range in different slices in the interest of realizing local accurate measures of the fluctuations in each slice, so we fix the size of the time window to $t_w = 1000$. For each time slice we fit a linear model to each of the I runs of our simulation, then we compute the residuals as the difference between the linear model and the data from the simulations. We have I residuals that measure the size of the fluctuations at each time step, and we plot a range corresponding to twice their standard deviation to provide information about the size of the fluctuations at each time step.

III. PROBABILITY OF A DEFECTOR SURVIVING IN THE HARMONY GAME FOR HIGHER AVERAGE DEGREE

In Figure 4 we show the probability of a defector surviving in a full-cooperative population, calculated numerically using equation 15, for the case of a higher average degree, $\langle k \rangle = 20$, than in the main text. The main impact of an increased value of average degree is a significant decrease of the probability, for any number of layers, or values of S and T. The effects discussed in the main text remain for this case too, but attenuated (note that the range of values for the probability are smaller in this case). In general, the probability increases with T, but it is only slightly dependent of S. As the number of layers increases, the probability becomes more uniform in the $S-T$ plane, increasing in general.

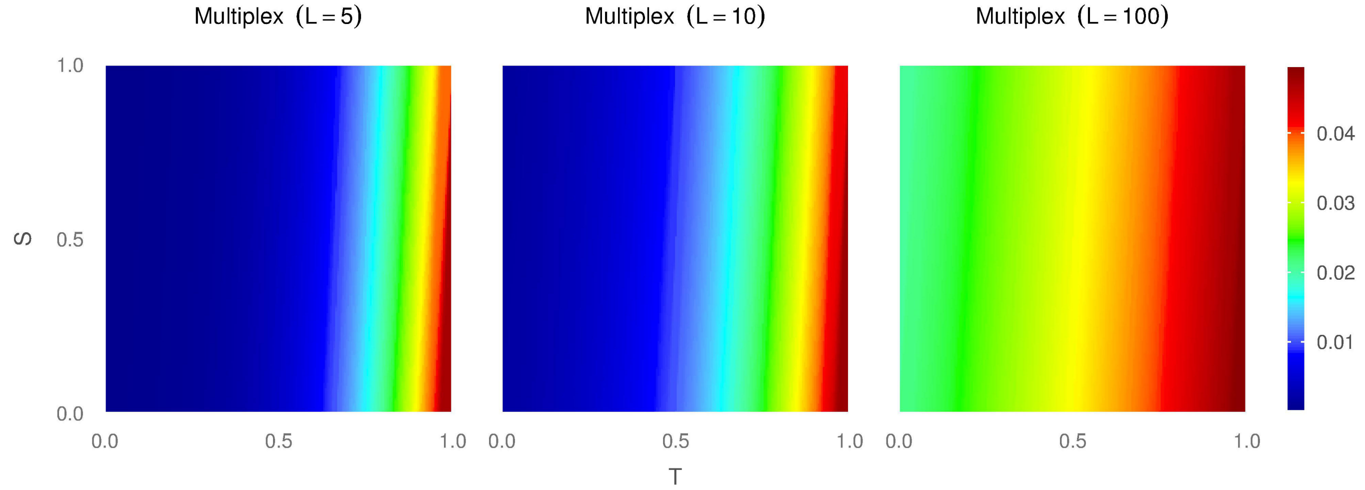

FIG. 4: Probability of a defector surviving in the Harmony Game for 5 layers (left), 10 layers (middle) and 100 layers (right) for an average connectivity of $\langle k \rangle = 20$, calculated according to equation 15 from the main text.

IV. PERCENTAGE OF COOPERATION AMONG MIXED INDIVIDUALS, AND PAYOFF OF COOPERATORS AND DEFECTORS

In this final section, we address the analysis of the percentage of cooperation among mixed individuals, as well as the payoff obtained by both cooperators and defectors. Regarding the former, we observe that the percentage of mixed individuals playing as cooperators is very high in the Harmony game, and in the upper diagonal of the Stag-Hunt game, as well as the upper diagonal of the Snow-Drift. In the Prisoner's Dilemma game, however, it is zero except for a small region near the weak limit, when cooperation is relatively inexpensive. This general situation gets emphasize by the increasing of the number of layers.

Regarding the latter, we observe that the average payoff among cooperators in obviously the highest in the Harmony game, and upper diagonals of both Stag-Hung and Snow-Drift, and it is zero in the hardest, bottom diagonal of the Prisoner's Dilemma game, with a wide transition area of intermediate values separating both regions. Moreover, this description seems to be independent of the number of layers in the system. Finally, the only regions where defection gets a moderate payoff are within the Snow Drift game, while it is near zero anywhere else. This picture is also independent of the number of layers.

V. INITIAL FRACTION OF COOPERATORS.

For completeness, in Fig. 6 we show the stationary average fraction of cooperation for the four-game plane and various numbers of layers, for three different initial fractions of cooperators (upper row is $c_0 = 0.25$, middle row is $c_0 = 0.5$ and bottom row is $c_0 = 0.75$). We will briefly discuss now the differences between the previously explained case of $c_0 = 0.5$, and the other two scenarios.

We observe that, for a given game quadrant and a given number of layers, increasing the initial fraction of cooperation has in general a positive but moderate impact on the stationary fraction of cooperation, specially in the

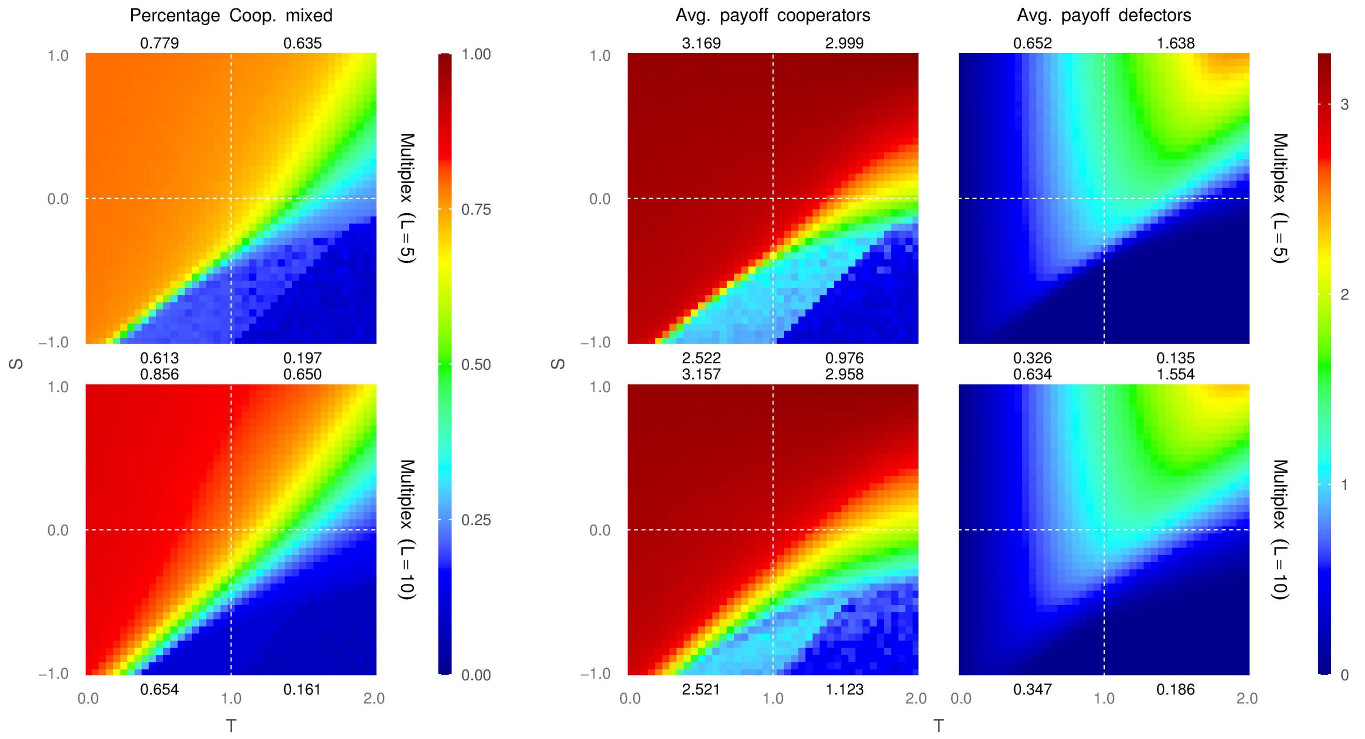

FIG. 5: Percentage of cooperation in mixed individuals (left column), average direct payoff obtained playing as cooperator (middle column) and average direct payoff obtained playing as defector (left column) for 5 layers (top row) and 10 layers (bottom row) multiplex, in the four games. The corresponding averaged values over the quadrants are also provided (upper-left is the Harmony Game, upper-right is the Snow Drift, Stag-Hunt is the lower-left, and the Prisoner's Dilemma in the lower-right).

Stag Hunt and Prisoner's Dilemma games. In the former one, we have an unstable evolutionary equilibrium in mixed populations, so the change of c_0 has a significant impact on the final outcome. In the case of Prisoner's dilemma game, an increase in the initial fraction of cooperators means an increase in the probability that clusters of cooperators forms.

The effect discussed in this paper when adding layers to the system still holds or is even emphasized by an increased initial fraction of cooperators: the overall stationary value of cooperation increases with the number of layers in the Prisoner's Dilemma Game and Stag-Hunt, the region of coexistence between both strategies widens for the Snow-Drift Game, and the Harmony game presents a small decrease of cooperation.

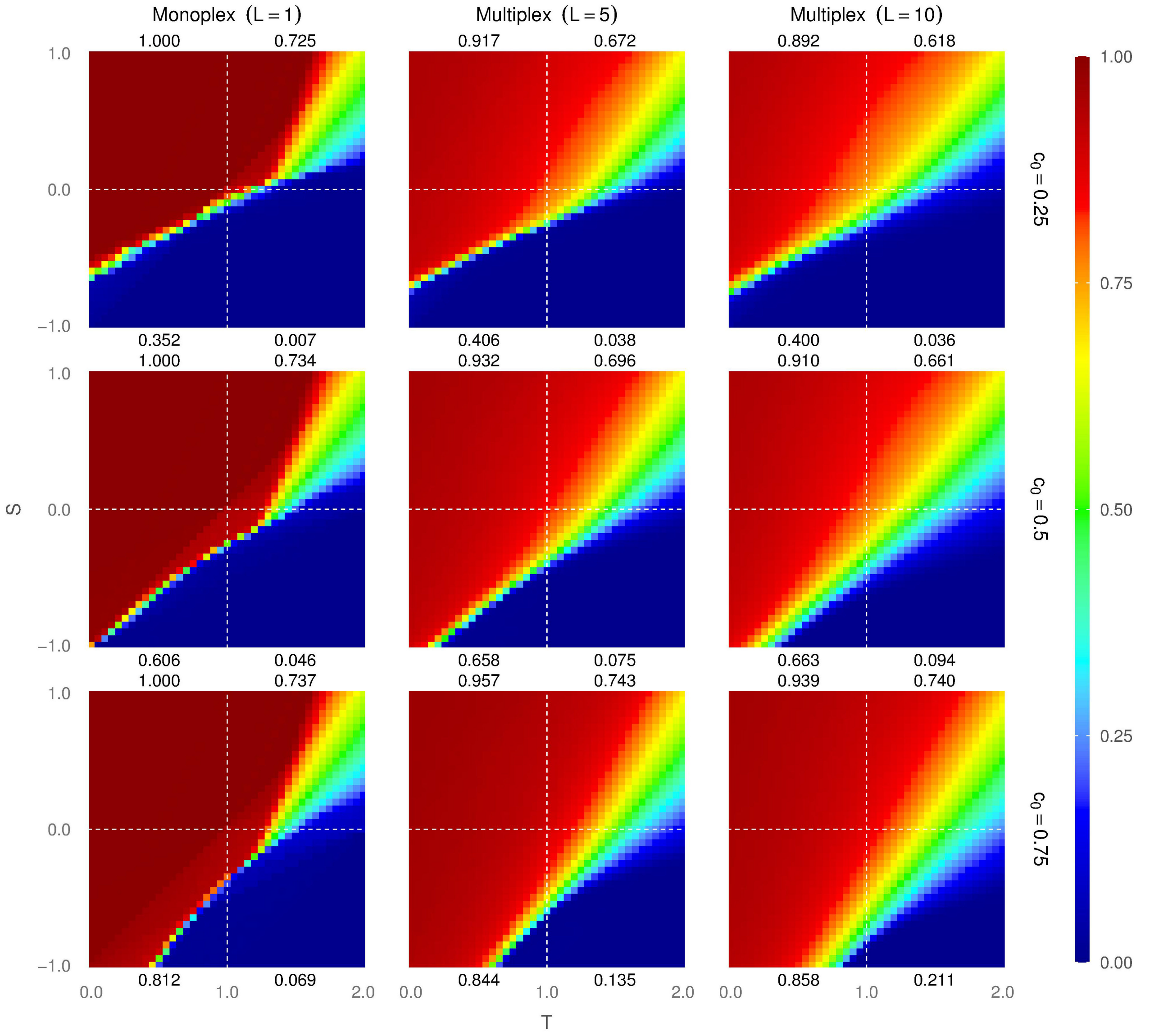

FIG. 6: Asymptotic density of cooperators $\langle c \rangle$ for networks with different number of layers ($L = 1$ in the left column, $L = 5$ in the central column, $L = 10$ in the right column), and different initial fraction of cooperation ($c_0 = 0.25$ in the top row, $c_0 = 0.5$ in the central row, $c_0 = 0.75$ in the bottom row). The plane $T - S$ is divided into four major regions that correspond to the four games under study: the upper-left area is the Harmony Game, the upper-right is the Snow Drift, Stag-Hunt is in the lower-left, and the Prisoner's Dilemma is in the lower-right. The average asymptotic density of cooperators for each one of the games is also indicated, as a numerical value, next to the corresponding quadrant.
